# Lycopene ameliorates oxidative stress in the aging chicken ovary via activation of Nrf2/HO-1 pathway

**DOI:** 10.18632/aging.101526

**Published:** 2018-08-16

**Authors:** Xingting Liu, Xin Lin, Siyu Zhang, Changquan Guo, Jian Li, Yuling Mi, Caiqiao Zhang

**Affiliations:** 1 Department of Veterinary Medicine, College of Animal Sciences, Zhejiang University, Hangzhou 310058, China

**Keywords:** lycopene,ovarian aging, oxidative stress, Nrf2/HO-1, chicken

## Abstract

After 480 days of age, high-producing hens are likely to be subject to ovarian aging, mainly due to oxidative stress. In this study, the amelioration of ovarian aging in chickens, using a plant antioxidant, lycopene, was investigated. The activity of the Nrf2/HO-1 pathway in chicken ovaries at different ages (90, 150, 280 and 580 days old) were compared to elucidate any age-related changes. Subsequently, the putative attenuating effect of lycopene (100 ng/mL) on ovarian aging was evaluated through the establishment of a D-gal-induced aging ovarian culture model. The cultured ovarian tissues of young (280 days) and old (580 days) hens were treated with lycopene for 72 h to verify protective effects of lycopene on naturally aged ovaries. Results showed that the Nrf2/HO-1 pathway was down-regulated during the ovarian aging process. Lycopene rescued the decreased antioxidant capacity by increasing the activities of antioxidases and activating the Nrf2/HO-1 pathway in both D-gal-induced and naturally aged ovaries. Moreover, lycopene promoted cell proliferation and inhibited apoptosis in both D-gal-induced and naturally aged ovaries. Lycopene also alleviated D-gal-induced mitochondrial damage in the living granulosa cells. In conclusion, lycopene can effectively ameliorate the oxidative stress in aging hen ovaries via the activation of the Nrf2/HO-1 pathway.

## Introduction

Overt signs of aging occur in the ovaries both earlier and more rapidly than in any other organ. Female fecundity is negatively correlated with increasing chronological age [1]. Ovarian aging is characterized by an age-related gradual decrease in both the quantity and the quality of oocytes. Poor oocyte quality is the major age-related contributing factor responsible for the decline in female fertility [2,3]. In mammals, the decline in oocyte quality is also known to be a major cause of aneuploidy, miscarriages and birth defects [4,5]. In domestic chickens, decline in egg production also occurs with advancing age which causes a great loss of income to the poultry industry [6].

Among all the inducing factors of ovarian aging, oxidative stress, caused by the accumulation of reactive oxygen species (ROS) generated during metabolic activity, is one of the most dominant factors [7–9]. Physiological ROS levels are considered to act to maintain the normal signal transduction pathways in folliculogenesis, oocyte maturation and ovulation [10], but high ROS levels induce oxidative damage [11]. Growing evidence has shown that oxidative stress caused by excessive ROS leads to the damage of oocytes and granulosa cells within follicles. Studies have demonstrated that ROS accumulation in the ovaries leads to antral follicle destruction and oocyte dysfunction in mice [12,13]. In the rat, oxidative stress has been shown to induce granulosa cell apoptosis and antral follicles atresia [14]. Numerous other studies have demonstrated that oxidative stress is associated with granulosa cell dysfunction and apoptosis and in an age-related decline in female fertility [15,16]. As in mammals, hens also undergo severe decreases in fecundity due to the increase in ovarian oxidative stress during the aging process [17].

In normal cells, there is a complex antioxidant system which makes use of antioxidant enzymes and biological antioxidants. However, the ability of the antioxidant system in the ovary to scavenge ROS decreases severely during the aging process [18]. Hence, a variety of edible compounds containing antioxidants have been screened relating to their efficacy in aiding in the protection of cells against oxidative stress. The antioxidant effects of these food based compounds are exerted either by their direct scavenging of free radicals or by their action of increasing the endogenous cellular antioxidant potential indirectly, via the activation of related signaling pathways. Nuclear factor erythroid 2-related factor 2 (Nrf2) is well established as a critical transcription factor that regulates antioxidant genes and is responsible for the induction of various cellular defense mechanisms against oxidative stress. Under normal conditions, Nrf2 is localized in the cytoplasm and is sequestered by its repressor kelch-like ECH-associated protein1 (Keap 1). Under conditions of oxidative stress, Keap1 alters its conformation, thus becoming no longer able to bind Nrf2 molecules. In this case, Nrf2 accumulates and enters the nucleus and activates the transcription of its target genes [19]. Nrf2 can promote the expression of antioxidative enzymes such as the hemeoxygenase-1 (HO-1), which prevents cellular apoptosis [20,21]. Salvianolic acid A can act to protect RPE cells against oxidative stress through the activation of Nrf2/HO-1 signaling [22]. However, as a reflection of tissue antioxidant status, the expression of Nrf2 and its downstream HO-1 in ovaries remains yet to be elucidated and compared from hens of different ages.

The establishment of aging models is an effective method for the study of many aging mechanisms. D-galactose (D-gal) is a reducing sugar, which generates advanced glycation end products (AGEs) in the oxidative metabolism *in vivo*. Animals treated with D-gal can be used to mimic natural aging [23]. In rodents, the D-gal-induced aging model has been widely used for the exploration of aging mechanisms and in the screening for the anti-aging substances [24,25].

In recent years, many natural plant extracts such as resveratrol [26], hesperidin [27] and grape seed proanthocyanidin extract [17] have been applied towards reducing oxidative stress in the ovaries in order to maintain normal function. Lycopene is a kind of carotenoid compound that exists in tomatoes, watermelon, pink grapefruit, guava and other red fruit [28]. It possesses an extremely effective ability to scavenge free radicals and provides protections against oxidative damage in various tissues [29]. Dai et al. demonstrated that lycopene attenuates colistin-induced nephrotoxicity via the activation of the Nrf2/HO-1 pathway in mice [30]. Lycopene ameliorates atrazine-induced oxidative damage in the adrenal cortex by the activation of the Nrf2/HO-1 pathway [31]. However, the antioxidant role of lycopene has not been well elucidated in the senescent ovaries of the laying hens.

In the present study, the Nrf2/HO-1 pathway in hen ovaries at 90 (D90), 150 (D150), 280 (D280) and 580 (D580) days old was compared to elucidate the relationship between oxidative stress and the Nrf2/HO-1 pathway during the ovarian aging process. Subsequently, a D-gal-induced aging ovarian model was established to evaluate the protective effects of lycopene against ovarian oxidative stress during ovarian aging *in vitro*. Concurrently, cultured ovaries from D280 and D580 hens were treated with lycopene to verify the protective effects of lycopene on the ovarian oxidative stress resulting from the natural aging process. The results expand our knowledge about retarding ovarian aging in poultry and extending the laying periods of older hens.

## RESULTS

### Age-related changes in the activity of the Nrf2/HO-1 pathway

**Figure 1:**
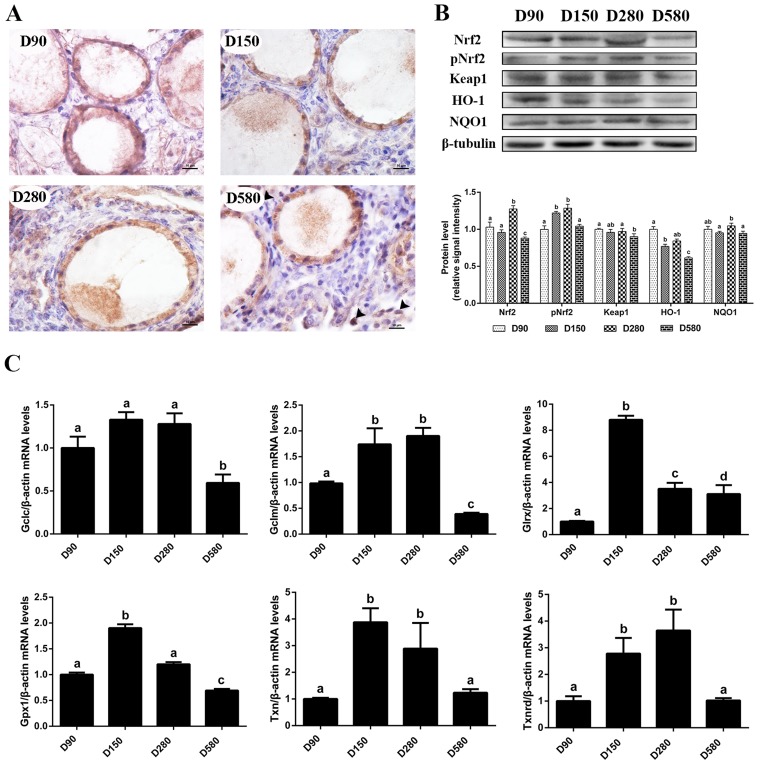
**Age-related changes in the activity of the Nrf2/HO-1 pathway.** (**A**) Immunohistochemistry of Nrf2 in the ovaries of hens aged 90, 150, 280 and 580 days, scale bar: 10 μm, black arrowheads: Nrf2 located in the nucleus. (**B**) Age-related changes in relative expression levels of Nrf2, pNrf2, Keap1, HO-1 and NQO1. (**C**) Age-related changes in transcription levels of Nrf2/HO-1 downstream genes: *Gclc*, *Gclm*, *Glrx*, *Gpx1*, *Txn*, *Txnrd*. Values are expressed as the means±s.e.. The relative abundance of each transcript was normalized to a *β*-actin and expressed as fold change over D90 ovaries. Different lowercase letters indicate significant differences (*P* < 0.05).

In a previous study we reported that the ovarian antioxidant capacity decreased significantly in hens during the aging process as a result of decreased antioxidase and transcription of antioxidant genes as well as increased oxidant levels in ovarian tissues [17]. In the present study, the expression of proteins related to the Nrf2 pathway and the related downstream genes were determined. The results of immunohistochemical staining of the ovaries showed that the Nrf2 protein was predominantly located in the cytoplasm from D90 to D280. However, Nrf2 expression was reduced in the cytoplasm and had trans-located to the nuclei in D580 hen ovaries (Fig. 1A). In addition, western blot analysis confirmed that the expression of Nrf2, phosphorylated Nrf2 (pNrf2) and HO-1 proteins were all down-regulated significantly at D580 compared with their expression in D150 and D280 hens. There were no consistent changes of any significance in either the expression of Keap1 or the expression of NADPH: quinone oxidoreductase 1 (NQO1) in D580 hen ovaries as compared with ovaries from younger ovaries (Fig. 1B). The mRNA abundance of *Gclc*, *Gclm*, *Gpx1*, *Txn* and *Txnrd* in the ovarian tissues increased from pullets to laying hens then gradually decreased during the subsequent aging process. The mRNA abundance of *Gclc*, *Gclm* and *Gpx1* in D580 ovaries was significantly lower than those of the other three stages. The transcription of *Txn* and *Txnrd* were markedly lower in D90 and D580 ovaries than that in D150 and D280 ovaries. Interestingly, the expression of *Glrx* in D580 ovaries was markedly lower than those in the D150 and D280 ovaries while it was higher than in D90 ovaries (Fig. 1C). These results suggested that the Nrf2/HO-1 pathway had been down-regulated during the ovarian aging process in hens.

### Effects of lycopene on the morphological and ultrastructure changes of the D-gal-induced aged ovarian tissues

**Figure 2:**
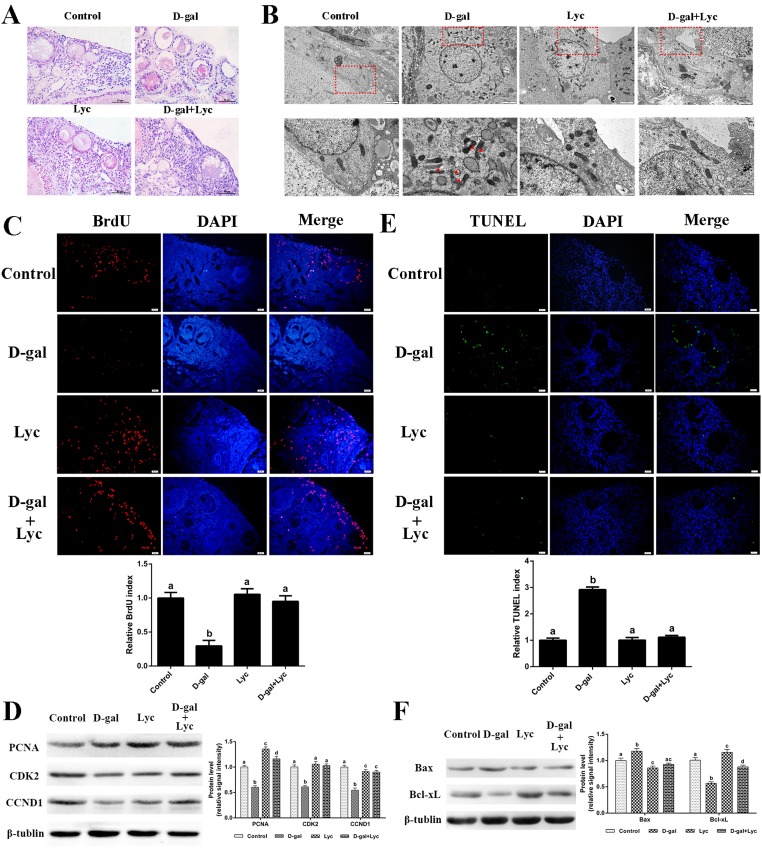
**Protective effects of lycopene on D-gal-induced aged ovaries.** (**A**) Effect of lycopene on D-gal-induced morphological changes of ovarian tissues, scale bar: 50 µm. (**B**) Effect of lycopene on D-gal-induced ultrastructural changes of granulosa cells: the four pictures in the lower row are the higher magnifications of the red squares from the four pictures in the upper row, respectively, scale bar: 2 µm (upper); 1 µm (lower), red arrowheads: fragmented mitochondria. (**C**) Effect of lycopene on D-gal-induced decline of BrdU index, scale bar: 20 µm; (**D**) Relative expression of proteins related to cell proliferation. (**E**) Effect of lycopene on D-gal-induced increase of TUNEL index, scale bar: 20 µm. (**F**) Relative expression of proteins related to pro-apoptosis (Bax) and anti-apoptosis (Bcl-xL). Values are expressed as the means±s.e.. Different lowercase letters indicate significant differences (*P* < 0.05).

In order to study the attenuating effects of lycopene on ovarian aging, an *in vitro* D-gal-induced ovarian aging model was established. HE staining showed that treatment of ovarian tissues with 2.5 mg/mL D-gal for 72 h obviously induced the apoptosis of the granulosa cells [17]. In contrast to those of the control group, the structure of the growing follicles in D-gal-induced aged ovarian tissues was damaged and displayed a loose and irregular arrangement of the granulosa cells. These adverse changes were all alleviated by the combined treatment of lycopene but treatment of 100 ng/mL lycopene for 72 h alone had no obvious effect on morphology of the granulosa cells and growing follicles (Fig. 2A). These results demonstrate that the impairment of the granulosa and growing follicles in D-gal-induced aged ovarian tissues could be reversed by lycopene *in vitro*.

In contrast to the control group, the mitochondria in the living granulosa cells of the D-gal-induced aged ovarian tissues were fragmented and swollen. As expected, the phenomenon of mitochondrial fragmentation and swelling in living granulosa cells was alleviated after 72 h simultaneous treatment with lycopene and D-gal. Meanwhile, no obvious difference was found in mitochondrial morphology between lycopene treatment and the control groups (Fig. 2B). These data indicated that lycopene could partially rescue the D-gal-induced ultrastructural damages of the living granulosa.

### Effects of lycopene on the somatic cell proliferation decline in the D-gal-induced aged ovarian tissues

D-gal treatment exerted a dose-dependent detrimental effect on ovarian somatic cell proliferation [17]. Treatment with 2.5 mg/mL D-gal alone decreased the BrdU index remarkably while lycopene treatment alone did not result in any change in the BrdU index. In addition, the decline of the BrdU index, as induced by D-gal, was inhibited by lycopene supplementation (Fig. 2C). D-gal remarkably decreased the expression of PCNA and CDK2, whilst lycopene reversed these alterations. The expression of PCNA in the D-gal and lycopene treatment group was significantly higher than the control group. D-gal decreased the expression of CCND1 and lycopene partially rescued this decrease. However, this decrease was not restored for the control group. Interestingly, treatment with lycopene alone significantly increased the expression of PCNA but not CDK2 or CCND1 (Fig. 2D). These data suggested that the decline of somatic cell proliferation in the D-gal-induced aged ovarian tissues was inhibited by lycopene supplementation.

### Effects of lycopene on cell apoptosis in the D-gal-induced aged ovarian tissues

The results from the TUNEL assay showed that D-gal treatment significantly increased the TUNEL index of the ovarian tissues, while lycopene supplementation reversed this increase. Treatment with lycopene alone did not change the TUNEL index (Fig. 2E). Western blot analysis of the apoptosis-related proteins showed that the expression of Bax increased significantly, while the expression of Bcl-xL decreased remarkably, in the D-gal-induced aged ovarian tissues as compared to the corresponding levels in the control group. Consistent with expectations, the changes in the expression of Bax and Bcl-xL were both normalized by simultaneously supplementation with lycopene. Meanwhile, treatment with lycopene alone decreased Bax expression and increased Bcl-xL expression (Fig. 2F). These results suggest that the increase of cell apoptosis in the D-gal-induced aged ovarian tissues was prevented by lycopene supplementation.

### Effects of lycopene on the antioxidant capacity decline in the D-gal-induced aged ovarian tissues

**Figure 3:**
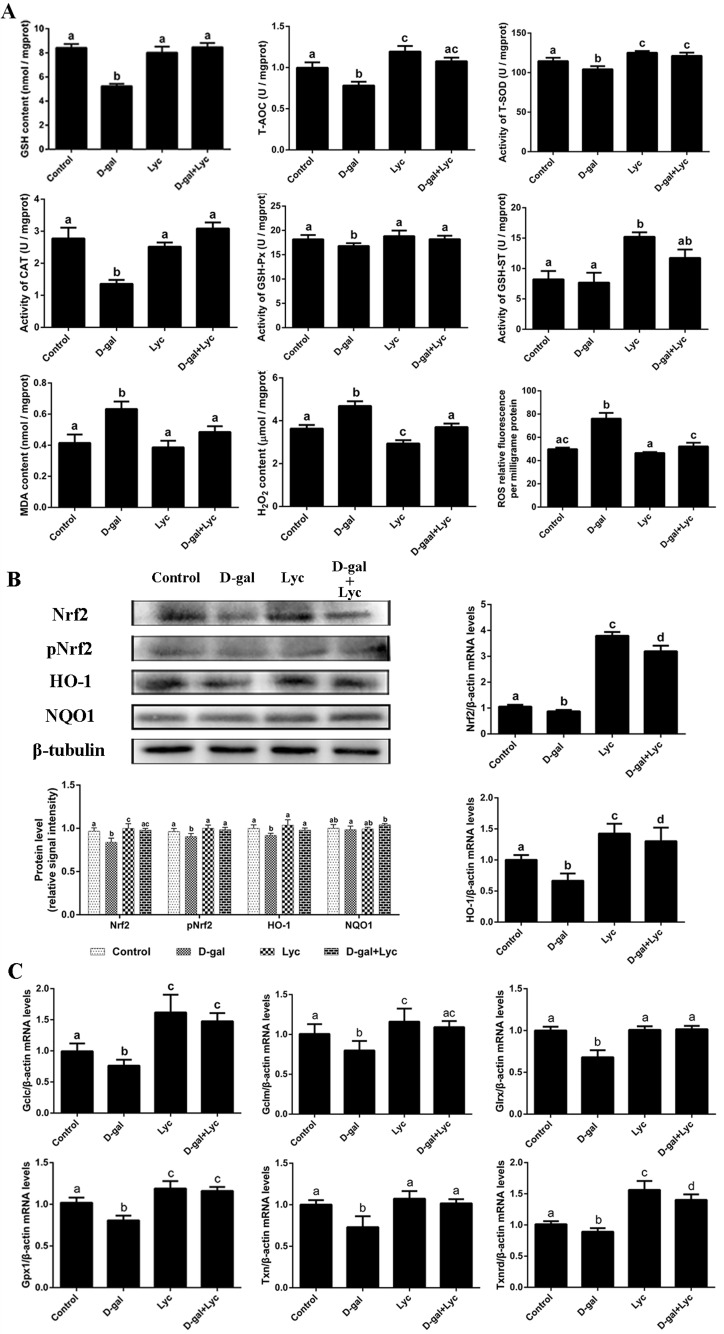
**Effects of lycopene on decreased antioxidant status in D-gal-induced aged ovarian tissues and the activities of Nrf2/HO-1 pathway.** (**A**) Effect of lycopene on decreased antioxidants status in the D-gal-induced aged ovarian tissues. (**B**) Effect of lycopene on the down-regulated expression of Nrf2, pNrf2 and HO-1, and the mRNA abundance of *Nrf2* and *HO-1*. (**C**) Effect of lycopene on down-regulated mRNA abundance of Nrf2/HO-1 downstream genes. Values are expressed as the means±s.e.. Different lowercase letters indicate significant differences (*P* < 0.05).

In order to clarify the effect of lycopene on the D-gal-induced aged ovarian tissues, the activities of antioxidant enzymes as well as the contents of malonaldehyde (MDA), hydrogen peroxide (H_2_O_2_) and ROS in ovarian tissues were measured from the four groups. The results showed that the glutathione (GSH) contents and the total antioxidant capacity (T-AOC) in the D-gal-induced ovarian tissues were remarkably lower than those of the control group. Meanwhile, the activities of total superoxide dismutase (T-SOD), catalase (CAT) and glutathione peroxidase (GSH-Px) decreased significantly after 72 h treatment with D-gal. These descending changes were all attenuated by simultaneous supplementation with lycopene. However, neither treatment with D-gal alone, or D-gal combined with lycopene, had any influence the activity of glutathione S-transferase (GSH-ST). Meanwhile, T-AOC and the activity of T-SOD had increased markedly compared with control group levels after treatment with lycopene alone for 72 h. MDA, H_2_O_2_ and ROS levels also increased significantly in the D-gal-induced aged ovarian tissues compared with equivalent levels in the control group, while simultaneous administration of lycopene prevented these increases. Furthermore, 72 h treatment with lycopene alone remarkably decreased the contents of H_2_O_2_ in the ovarian tissues (Fig. 3A). These results indicated that the decline in the antioxidant capacity of the D-gal-induced aged ovarian tissues was prevented by simultaneous supplementation with lycopene.

### Effects of lycopene on the down-regulation of the Nrf2/HO-1 pathway in D-gal-induced aged ovarian tissues

To evaluate the effects of D-gal alone, or D-gal combined with lycopene, on the Nrf2/HO-1 pathway in ovarian tissues, the expression of Nrf2, pNrf2, HO-1 and NQO1, and the mRNA abundance of *Nrf2*, *HO-1* and their related downstream genes were determined. The results showed that in the D-gal-induced aged ovarian tissues, the expression of Nrf2, pNrf2 and HO-1 had decreased significantly while the expression of NQO1 had not changed remarkably. Simultaneous treatment with lycopene prevented this decline in the expression of Nrf2, pNrf2 and HO-1 effectively. Meanwhile, the mRNA abundance of *Nrf2* and *HO-1* had both been markedly down-regulated in the D-gal-induced aged ovarian tissues, where lycopene supplementation was able to effectively suppress these declines. Interestingly, compared with the control group, treatment with either lycopene alone or lycopene combined with D-gal, both significantly increased the mRNA abundance of *Nrf2* and *HO-1* (Fig. 3B). The mRNA abundance of *Gclc*, *Gclm*, *Gsr*, *Gpx1*, *Txn* and *Txnrd* had decreased remarkably compared to that of the control, however, these descending changes were all prevented by simultaneous lycopene supplementation. The transcription of *Gclc*, *Gclm*, *Gpx1* and *Txnrd* in ovarian tissues that had been treated with lycopene alone or lycopene combined with D-gal were higher than those of the control group (Fig. 3C). These data implied that the inhibitory effects of the D-gal-induced aging on Nrf2/HO-1 pathway were suppressed by lycopene supplementation.

### The oxidative stress prevention effect of lycopene on D-gal-induced aged ovaries was dependent on the induction of the Nrf2/HO-1 pathway

**Figure 4:**
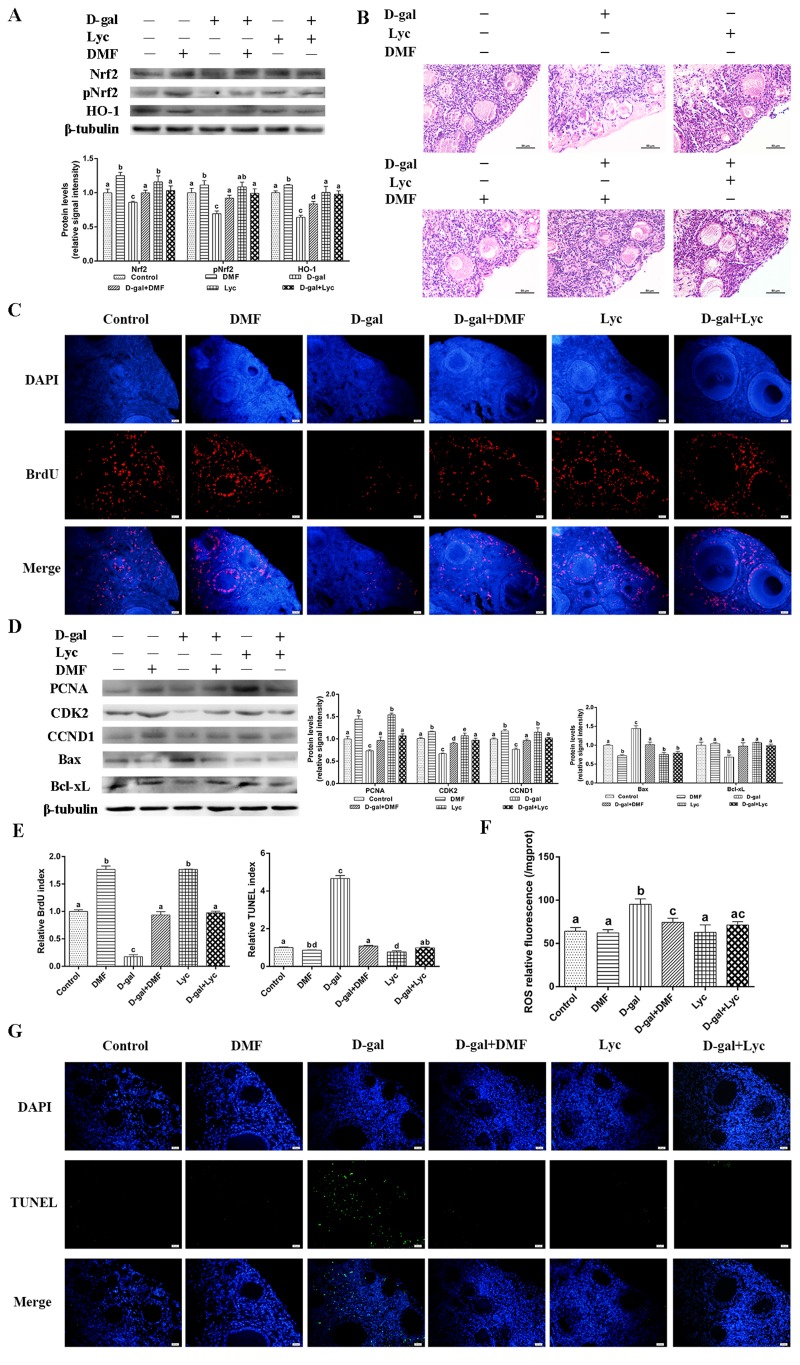
**Protective effect of lycopene on oxidative stress in aged ovarian tissues was similar to the effects of DMF.** (**A**) Relative changes in the expression of Nrf2, pNrf2 and HO-1 after treatment with D-gal alone or combined with DMF or lycopene. (**B**) Changes in the morphology of ovarian tissues after treatment with D-gal alone or combined with DMF or lycopene, scale bar: 50 µm. (**C** and **E**) Changes in BrdU index in ovarian tissues after treatment with D-gal alone or combined with DMF or lycopene, scale bar: 20 µm. (**D**) Relative changes in the expression of proteins related to cell proliferation and cell apoptosis in ovarian tissues after treatment with D-gal alone or combined with DMF or lycopene. (**E** and **G**) Changes in TUNEL index in ovarian tissues after treatment with D-gal alone or combined with DMF or lycopene, scale bar: 20 µm. (**F**) Changes in ROS levels of the ovarian tissues after treatment with D-gal alone or combined with DMF or lycopene. Values are expressed as the means±s.e.. Different lowercase letters indicate significant differences (*P* < 0.05).

To characterize the molecular mechanisms underlying the inhibitory effects of lycopene on oxidative stress in the aged ovaries, the Nrf2/HO-1 pathway was activated and inhibited by the activator (dimethyl fumarate, DMF) [32] and the antagonist (ML385) [33], respectively. Results showed that treatment with lycopene alone intensively induced Nrf2, pNrf2 and HO-1 expression, in a manner similar to DMF, an Nrf2 activator. In addition, the decline in the expression of Nrf2, pNrf2 and HO-1 were normalized by the administration of lycopene as well as with DMF (Fig. 4A). HE staining showed that the structural damage of both granulosa cells and growing follicles in the D-gal-induced aged ovarian tissues were all rescued by lycopene supplementation as well as with DMF. Treatment with lycopene or DMF alone did not change the morphology of the growing follicles and granulosa cells (Fig. 4B). The BrdU index and the expression of PCNA, CDK2 and CCND1 were remarkably higher in the lycopene and DMF groups. Meanwhile, the inhibitory effects of D-gal on the BrdU index and expression of PCNA, CDK2 and CCND1 were all reversed by lycopene as well as by DMF (Fig. 4C-E). The TUNEL assay showed that lycopene and DMF both decreased the TUNEL index significantly compared with that of the control. In addition, the increase of the TUNEL index in the D-gal-induced aged ovarian tissues was suppressed by either lycopene or DMF administration (Fig. 4E,G). Consistent with the results of the TUNEL assay, the expression of Bax in lycopene and DMF groups were markedly lower than those of the control and the up-regulation of Bax expression in D-gal-induced aged ovarian tissues was inhibited by lycopene or DMF supplementation. The down-regulation of Bcl-xL expression in D-gal-induced aged ovarian tissues was normalized by lycopene or DMF administration while treatment with lycopene or DMF alone did not change the Bcl-xL expression (Fig. 4D). Furthermore, D-gal treatment resulted in increased ROS levels in ovarian tissues, and lycopene and DMF supplementation both inhibited this increase (Fig. 4F). These data demonstrated that the protective effect of lycopene on aging ovarian tissues from oxidative stress was similar to the effect of DMF, a known Nrf2 activator.

**Figure 5:**
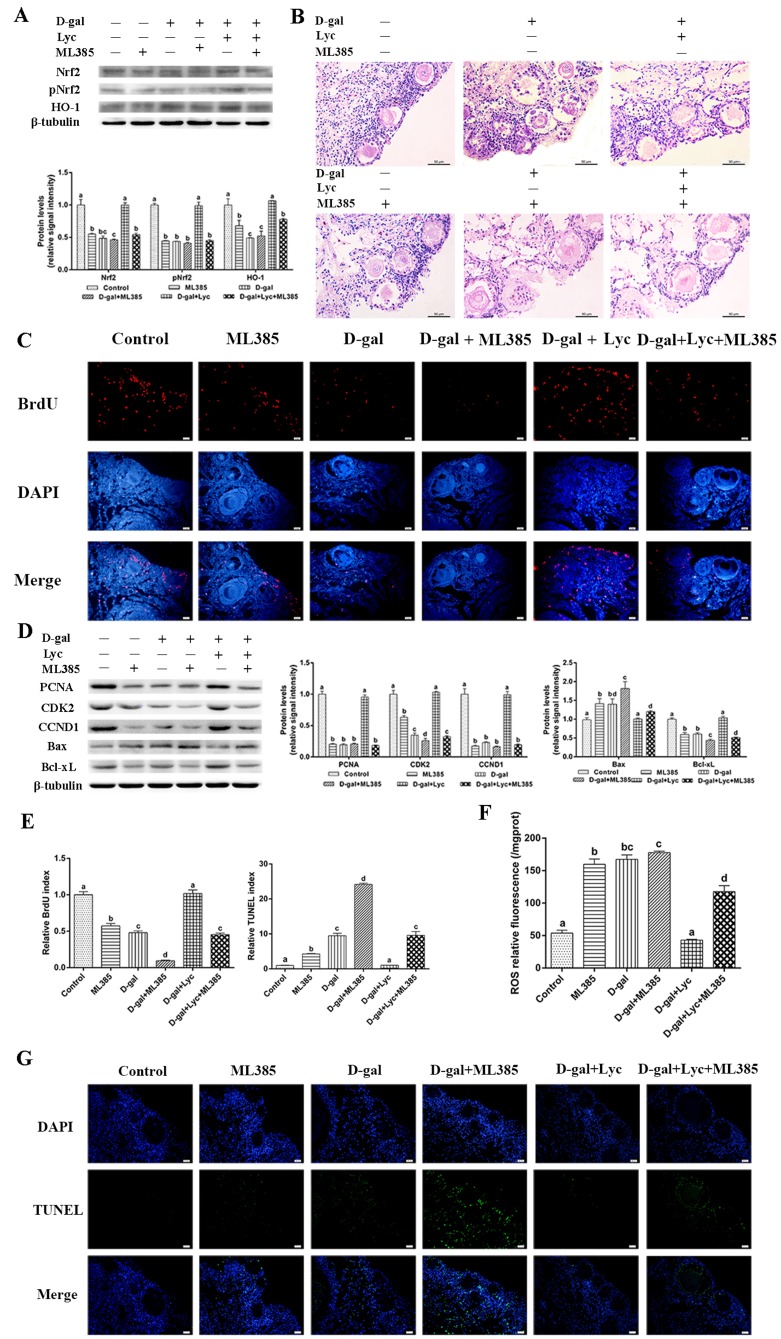
**Protective effect of lycopene on the oxidative stress in the aging ovarian tissues was abolished by ML385.** (**A**) Relative changes in the expression of Nrf2, pNrf2 and HO-1 after treatment with D-gal, ML385 alone or combined with lycopene. (**B**) Changes in the morphology of ovarian tissues after treatment with D-gal, ML385 alone or combined with lycopene, scale bar: 50 µm. (**C** and **E**) Changes in BrdU index in ovarian tissues after treatment with D-gal, ML385 alone or combined with lycopene, scale bar: 20 µm. (**D**) Relative changes in the expression of proteins related to cell proliferation and cell apoptosis in ovarian tissues after treatment with D-gal, ML385 alone or combined with lycopene. (**E** and **G**) Changes in TUNEL index in ovarian tissues after treatment with D-gal, ML385 alone or combined with lycopene, scale bar: 20 µm. (**F**) Changes in the levels of ROS in ovarian tissues after treatment with D-gal, ML385 alone or combined with lycopene. Values are expressed as the means±s.e.. Different lowercase letters indicate significant differences (*P* < 0.05).

To further elucidate the role of Nrf2 in the protective effects of lycopene on ovarian aging, we inhibited the Nrf2/HO-1 pathway using ML385. After 72 h treatment with ML385, significant decreases in expression of Nrf2, pNrf2 and HO-1 were observed in the ovarian tissues treated with or without D-gal and lycopene in comparison to the ovarian tissues treated without ML385 (Fig. 5A). Either D-gal or ML385 treatment damaged the structure of the growing follicles and induced granulosa cell apoptosis. In addition, the protective effect of lycopene on the D-gal-induced morphological damage was terminated by ML385 (Fig. 5B). The result of BrdU staining showed that the BrdU index in the ML385 group had decreased significantly as it also had in the D-gal-induced aged group. The attenuating effect of lycopene on the D-gal-induced decline in the BrdU index was also abolished by ML385 supplementation (Fig. 5C, E). Western blot analysis showed that the treatment with D-gal or ML385 alone or in combination for 72 h resulted in remarkably down-regulated the expressions of PCNA, CDK2 and CCND1. As expected, the inhibitory effect of lycopene on D-gal-induced decreases in the expressions of PCNA, CDK2 and CCND1 was blocked by ML385 treatment (Fig. 5D). Meanwhile, the result of the TUNEL assay showed that TUNEL index increased markedly with D-gal and ML385 treatment either alone or in combination. The protective effect of lycopene on the increase of the TUNEL index induced by D-gal was effectively inhibited by ML385 (Fig. 5E,G). In addition, similar to the D-gal-induced aged group, the expression of Bax increased significantly while the expression of Bcl-xL decreased significantly in the ML385 treatment group. The restoration of the D-gal-induced changes in the expression of Bax and Bcl-xL was blocked by ML385 simultaneous supplementation (Fig. 5D). Furthermore, D-gal and ML385 treatments, alone or in combination, resulted in increased ROS levels in the ovarian tissues. The attenuating effect of lycopene on the increase of ROS levels in the D-gal-induced aged ovarian tissues was suppressed by ML385 (Fig. 5F). These data suggested that the protective effect of lycopene on aged ovarian tissues was abolished by the Nrf2 antagonist, ML385.

### Lycopene is able to protect ovaries from oxidative stress *in vitro* during the natural aging process

**Figure 6:**
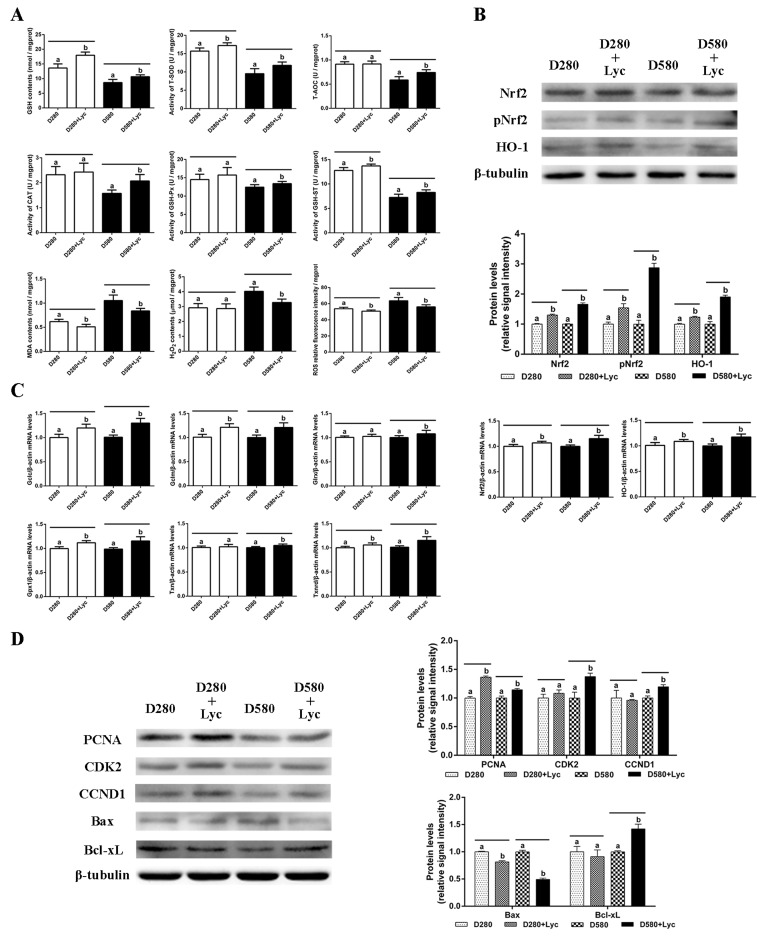
**Effects of lycopene on the antioxidant capacity, cell proliferation and apoptosis in the ovarian tissues of D280 and D580 hens *in vitro*.** (**A**) Effect of lycopene on antioxidant capacity in ovarian tissues of D280 and D580 hens *in vitro*. (**B**) Effect of lycopene on expression of Nrf2, pNrf2 and HO-1, and the mRNA abundance of *Nrf2* and *HO-1* in ovarian tissues of D280 and D580 hens *in vitro*. (**C**) Effect of lycopene on the mRNA abundance of Nrf2/HO-1 downstream genes in the ovarian tissues of D280 and D580 hens *in vitro*. (**D**) Effect of lycopene on the expression of proteins related to cell proliferation and apoptosis in the ovarian tissues of D280 and D580 hens *in vitro*. Each parameter was determined after 72 h of treatment with lycopene (100 ng/mL). Values are expressed as the means±s.e.. Different lowercase letters indicate significant differences (*P* < 0.05) for the same age.

To evaluate whether treatment with lycopene was able to protect naturally aging ovarian tissues from oxidative stress, D280 and D580 ovarian tissues were treated with / without lycopene for 72 h *in vitro*. The results showed that treatment with lycopene for 72 h *in vitro* significantly increased the GSH contents, T-AOC, and the activity of GSH-ST in both D280 and D580 ovarian tissues. Meanwhile, after treatment with lycopene, the activities of T-SOD, CAT and GSH-Px were increased remarkably in D580 but not in D280 ovarian tissues. In addition, lycopene treatment significantly decreased the MDA contents and ROS levels in both D280 and D580 ovarian tissues. However, lycopene supplementation only decreased the H_2_O_2_ contents in D580 but not D280 ovaries (Fig. 6A). These results indicate that the decline in ovarian antioxidant capacity that occurs during the natural aging process, could be prevented by lycopene supplementation *in vitro*.

The results of western blot analysis showed that lycopene treatment remarkably up-regulated the Nrf2, pNrf2 and HO-1 expression in both D280 and D580 ovaries. The mRNA abundance of *Nrf2* and *HO-1* had also increased significantly in D280 and D580 ovaries (Fig. 6B). In addition, the transcription of *Gclc*, *Gclm*, *Gpx1* and *Txnrd* in D280 and D580 ovaries were up-regulated remarkably compared to those of the control. However, lycopene treatment increased the mRNA abundance of *Glrx* and *Txnrd* only in D580, but not D280 ovaries (Fig. 6C). These data demonstrated that lycopene supplementation could activate the Nrf2/HO-1 pathway in the naturally aged ovaries.

After treatment with lycopene for 72 h *in vitro*, the expression of PCNA, CDK2 and CCND1 in D580 ovaries was increased markedly, while only the expression of PCNA in D280 ovaries was up-regulated significantly. Furthermore, lycopene supplementation remarkably down-regulated the expression of Bax and up-regulated the expression of Bcl-xL in the D580 hen ovaries. The Bax expression in D280 ovaries decreased significantly while Bcl-xL expression had not changed after lycopene supplementation (Fig. 6D). These results suggested that lycopene supplementation could maintain the homeostasis of cell proliferation and apoptosis in ovaries during the aging process.

## DISCUSSION

Female fertility is governed by the functional lifespan of the ovaries. This lifespan is mainly determined by the size of the oocyte reserve, something that has already been established prior to birth, as well as by the rate of endowment depletion [34,35]. Female fecundity is one of the first of the physiological functions negatively influenced by aging. Ovarian aging is accompanied by an age-dependent reduction in the ovarian follicle reserve and the decline in quantity and quality of the oocytes [2,3]. Growing evidence demonstrates that female mice and humans both exhibit an age-related decline in ovarian follicle reserve and oocyte quality [36]. One of the main causes of ovarian aging is oxidative stress that is induced by the gradual accumulation of ROS and an age-related decrease of the antioxidants in the ovary [8]. In a similar manner to mammals, a precipitous age-related decline in the egg production appears in laying hens, accompanied by a decrease in ovarian antioxidant capacity at the later laying stages [17]. Meanwhile, ovarian aging greatly shortens the ovarian functional lifespan and reduces the commercial values of the laying hens. However, there has been remarkably little focused study on ovarian aging mechanisms in laying hens. The elucidation of the mechanisms underlying ovarian aging and attenuation of oxidative stress may result in prolonging ovarian lifespans, and thus increasing laying performance.

**Figure 7:**
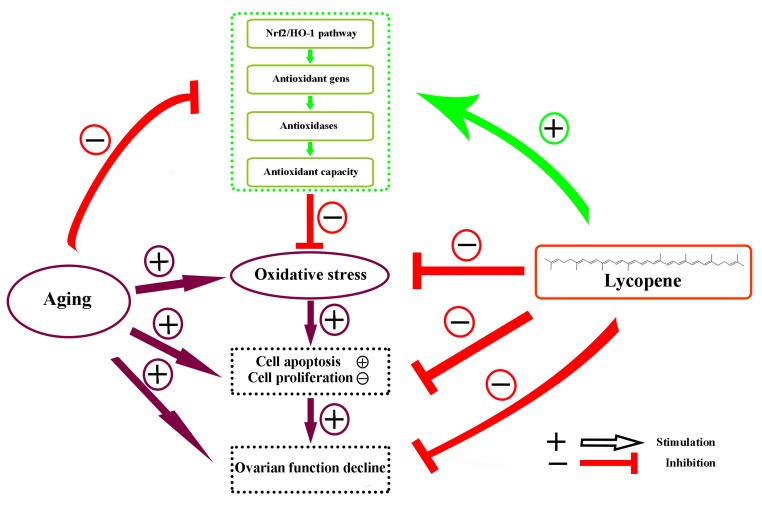
**Schematic diagram summarizing the mechanisms underlying the attenuating effect of lycopene against ovarian oxidative stress during the aging process in chickens.** The Nrf2/HO-1 pathway was down-regulated in the natural aging process in the laying hens. Lycopene attenuated the oxidative stress in aging ovaries via the activation of Nrf2/HO-1 pathway.

Accumulating evidence has supported the idea that the supplementation of edible antioxidants is an efficient measure to attenuate the oxidative stress in the ovary [37,38]. Lycopene is a member of carotenoid family of compounds found in tomatoes and other red fruits and vegetables. It has been shown to be a great scavenger of free radicals and a potential antioxidant attributing to the 11 conjugated bonds within the molecule [39,40]. As one of the most effective antioxidants found in plants, lycopene is widely used for protection against oxidative stress-mediated tissue injury. A previous study has demonstrated that lycopene protects cardiomyocytes from the oxidative damage of mtDNA induced by ischemia/reperfusion-injury in rats [41]. Orally administrated lycopene also attenuated diethylnitrosamine-induced hepatocarcinogenesis by modulating the Nrf-2/HO-1 and Akt/mTOR pathways in the rat [42]. Sahin et al. reported that lycopene activated antioxidant enzymes and a nuclear transcription factor system in heat-stressed broilers [43]. However, the antioxidant role of lycopene in senescent ovaries of the laying hens has not been clearly elucidated. In the present study, we investigated the effects of aging on the activity of the Nrf2/HO-1 pathway by comparing the expression of related proteins and genes in ovaries of hens in different laying stages. Then we investigated the protective effects of lycopene against oxidative stress in the D-gal-induced aged ovarian tissues. Furthermore, we verified the potential attenuation of lycopene on the oxidative stress in naturally aged ovaries. Our results indicated that the activity of the Nrf2/HO-1 pathway in the ovaries decreases significantly during natural aging. We showed that lycopene supplementation was able to effectively alleviate the oxidative stress in aged ovaries via the activation of the Nrf2/HO-1 pathways in laying hens (Fig. 7).

Nrf2 is a redox-sensitive transcription factor that confers cytoprotection against oxidative stress. The expression of *Cat*, *Sod* and *HO-1* and synthesis of GSH are all regulated by Nrf2 [44,45]. Activation of the Nrf2/HO-1 pathway could lead to increased levels of GSH, CAT and SOD in mouse kidneys [30,46]. Previous studies have demonstrated that Nrf2 expression and its target genes exhibit an age-dependent decrease [47,48]. In line with these findings, our results revealed that despite the increased translocation of Nrf2 protein from the cytoplasm to nuclei in D580 ovarian tissues compared with those of younger stages, however, down-regulation of Nrf2, pNrf2, HO-1 and their downstream genes appeared in D580 hen ovarian tissues. The reason for these results may be that Nrf2 remains inactive whilst in the nucleus. These data are in accordance with our previous study that the ovarian antioxidant status was decreased in the laying hens during the aging process [17].

The D-gal-induced aging model also relates to that of oxidative stress as there is a clear relationship between the activities of decreased antioxidases and the increased level of oxidants [24]. In the present study, we observed in the D-gal-induced aged ovarian tissues that the contents of GSH, T-AOC, and the activities of T-SOD, CAT and GSH-Px had decreased significantly, whereas the levels of MDA, H_2_O_2_ and ROS increased remarkably, as compared to the controls. In addition, D-gal treatment damaged the morphology of growing follicles and the granulosa cells as well as that of the mitochondria in the living granulosa cells. All these adverse changes were suppressed by the simultaneous supplementation of lycopene.

Apoptosis is an essential process for organ growth, development and the maintenance of normal homeostasis. However, excessive apoptosis caused by elevated intracellular ROS production may induce organ dysfunction [49]. Our results showed that in the D-gal-induced aged ovarian tissues, the TUNEL index and the expression of Bax increased significantly, while the expression of Bcl-xL decreased significantly. However, lycopene supplementation reversed these changes by increasing the Bcl-xL expression and decreasing the Bax expression. Meanwhile, BrdU incorporation and western blot analysis demonstrated the inhibition of somatic cell proliferation in the D-gal-induced aged ovarian tissues was suppressed by lycopene administration.

The activation of the Nrf2/HO-1 pathway upregulates the expression of many antioxidant genes and alleviates oxidative stress in the tissues [42]. Reichard et al. demonstrated that HO-1 induction by Nrf2 requires inactivation of the transcriptional repressor BACH1 [50]. The protective effect of lycopene against oxidative stress is via the activation of the Nrf2/HO-1 pathway [41–43]. Yang et al. reported that lycopene suppressed the activation of the TNFα-induced signaling pathway through upregulation of the Nrf2-mediated HO-1 expression in endothelial cells [51]. The Nrf2/HO-1 pathway represents the prime target for chemoprevention of cisplatin-induced nephrotoxicity by lycopene [52]. In this study, D-gal treatment significantly decreased the expression of Nrf2, pNrf2, HO-1 and the mRNA abundance of the *Nrf2*, *HO-1* and the downstream genes. These data were in line with the data *in vivo* that the Nrf2/HO-1 pathway was down-regulated during the ovarian aging process in laying hens. Simultaneous supplementation with lycopene rescued the descending changes, and treatment with lycopene alone up-regulated the expression of Nrf2, HO-1 and downstream *Gclc*, *Gclm*, *Gpx1* and *Txnrd* significantly, as compared to the controls. In order to elucidate the mechanism of the protective effect of lycopene related to oxidative stress, DMF and ML385 served as an activator and a small molecule inhibitor of Nrf2, respectively. These results showed that the protective effect of lycopene on aging ovarian tissues from oxidative stress was similar to the effect of DMF, whereas the protective effect of lycopene on aging ovarian tissues against oxidative stress was abolished by ML385 treatment.

The results of the verification experiments showed that lycopene improved the antioxidant capacity in D580 hen ovarian tissues *in vitro* and maintained the homeostasis between cell proliferation and apoptosis via the activation of the Nrf2/HO-1 pathway.

In summary, this study demonstrated that the Nrf2/HO-1 pathway was down-regulated in the natural aging process in the laying hens. Lycopene attenuated oxidative stress in both D-gal-induced aging and natural aging ovaries by the activation of the Nrf2/HO-1 pathway. This study provides first-hand evidence of the potential utilization of lycopene in the protection against ovarian aging in laying poultry. However, this study was conducted *in vitro*. The protective effects of lycopene on egg laying performance of older laying hens is still lacking. We aim to continue to explore the protective effects of lycopene on ovarian aging in laying hens through field studies.

## MATERIALS AND METHODS

### Reagents

D-gal was purchased from Aladdin Industrial Corporation (Shanghai, China); lycopene was from Sigma-Aldrich (St. Louis, USA). Dimethyl fumarate (DMF) and ML385 were from MedChemExpress (Shanghai, China). Antibodies against Nrf2 (ab31163), PCNA (ab29) and Bax (ab5714) were purchased from Abcam (Cambridge, UK). Antibodies against pNrf2 (ET1608-28) was purchased from Hangzhou HuaAn Biotechnology Co., Ltd. (Hangzhou, China). Antibodies against Keap1 (SC-365626), NQO1 (sc-271116) and β-Tubulin (sc-365791) were from Santa Cruz Biotechnology (Dallas, USA). Antibodies against Bcl-xL (BA0413), CDK2 (PB0562) and CCND1 (BA0770) were obtained from Boster Biological Technology Co. Ltd. (Wuhan, China). All other chemicals were purchased analytical grade.

### Animals and tissue culture

The Hyline brown hens (*Gallus domesticus*) used in this study were raised in a local farm and subjected to conventional feeding and management conditions. All animal experiments were performed in accordance with the recommendations in *the Animal Care and Use Guidelines* and were approved by *the Animal Care and Use Committee on the Ethics of Animal Experiments of Zhejiang University*. Sample collection was performed from 90 (D90), 150 (D150), 280 (D280) and 580 (D580) days old hens that reflected four different laying stages, Before laying, Early laying, Peak laying and Later laying periods. Hens were slaughtered by cervical bleeding post-anesthesia. Ovarian tissues without follicles of over 1 mm in diameter were collected for the following analyses.

For tissue culture, ovaries from D90 pullets were placed in ice-cold DMEM-F12. The ovarian cortex was dissected from the surface of the ovaries and cut into blocks (1-2 cm^3^). Each block was cultured on Millipore filters and placed into a well of a 24-well plate containing 500 μL complete DMEM-F12 supplemented with 5% chicken serum, 10 μg/mL insulin, 5 μg/mL transferrin, 30 nM selenite (Sigma-Aldrich), 100 mg/mL streptomycin and 100 U/mL penicillin. D-gal power was dissolved in DMEM-F12 medium directly. Lycopene was dissolved in medium containing 0.1% tetrahydrofuran (Sigma-Aldrich). All of the cultures were maintained in a humidified atmosphere with 5% CO_2_ at 38.5°C. The medium was replaced every 24 h. For senescence induction, a modified D-gal treatment protocol was used. Briefly, the cultured tissues were treated with D-gal in a gradient concentration from 1.25 mg/mL to 5 mg/mL to induce oxidative damage. Based on the evaluation of tissue morphology, cell proliferation and apoptosis rates [17], tissue fibrosis and antioxidant capacity, the dose of 2.5 mg/mL D-gal was chosen as the optimal concentration in the subsequent experiments (Supplementary Figure 1). Likewise, lycopene, in a gradient concentrations from 1 to 1000 ng/mL, was screened for its optimal concentration under D-gal induced stress. Based on the evaluation of tissue morphology and cell proliferation, 100 ng/mL lycopene was determined as the optimal concentration for the following formal experiments. The ovarian cortical blocks were divided randomly into four groups and were treated with D-gal (2.5 mg/mL), lycopene (100 ng/mL) and D-gal+lycopene for 72 h. After 48 h of culture, bromodeoxyuridine (BrdU, Sigma-Aldrich) was added into the complete medium at 20 μg/mL. After 72 h of treatment, ovarian tissues were collected for the subsequent determinations. Ovarian tissues for biochemical analysis, qRT-PCR and Western blot were cultured for 72 h without BrdU incorporation. For the activation and inhibition of the Nrf2, 3 μM DMF or 6 μM ML385 was supplemented into the medium, respectively, with minor adjustment according to the references [29,30].

### Morphological and ultrastructural observations

After 72 h culture, the ovarian blocks were fixed in 4% neutral paraformaldehyde solution for 24 h at 4°C, dehydrated in a grade ethanol, then cleared in xylene and embedded in paraffin. The embedded samples were sectioned at 5 μm and mounted slides. The paraffin sections of ovarian tissues were used for subsequent immunohistochemistry, BrdU and TUNEL detection. Hematoxylin and eosin (H&E) staining was performed using standard protocols.

The specimens of ovarian tissues were fixed with 2.5% glutaraldehyde in phosphate-buffered saline (PBS) for 24 h at 4°C after 72 h of culture. The specimens were then post-fixed with 1% Osmium tetroxide (OsO4) in PBS for 1.5 h at room temperature and rinsed in PBS. After dehydration in ascending concentration of ethanol and infiltration with a propylene oxide-Araldite mixture, the samples were embedded in Araldite. The blocks were sectioned using a Leica EM UC7 ultramicrotome (Leica Microsystems GmbH, Wetzlar, Germany) and the ultrathin sections were mounted on copper coated grids. The ultrathin sections were stained with uranyl acetate and alkaline lead citrate for 5 to 10 min. Finally, the cell ultrastructure was observed using a transmission electron microscope (Tecnai G2 Spirit 120KV FEI Company, Hillsboro, USA).

### Measurements of oxidative parameters

After the 72 h treatment *in vitro*, ovarian cortical blocks were hemogenized in PBS and then centrifuged at 800 *g* for 20 min at 4°C. The supernatants were used for the determination of total protein concentration and the measurements of the GSH, T-AOC, T-SOD, CAT, GSH-Px, GSH-ST, MDA and H_2_O_2_ according to the manufacturer’s instruction with kits (Nanjing Jiancheng Bioeng Ins, Nanjing, China). For the measurement of ROS levels, cultured tissues were digested into a single cell suspension and were then used for the determination of total protein concentration and the ROS levels according to the protocols with the ROS Assay Kit (Nanjing Jiancheng Bioeng Ins).

### Quantitative Real-time PCR analysis

Total RNA was isolated from cultured ovarian tissues using TRIzol (Takara, Shiga, Japan). 2 μg RNA was used for reverse transcription using a RevertAid First Strand cDNA Synthesis Kit (Thermo Fisher Scientific, San Jose, USA). Quantitative real-time polymerase chain reaction (qRT-PCR) was performed using SYBR^®^ Premix Ex Taq^TM^ Kit (Takara) on an ABI 7500HT Real-time PCR detection system (Applied Biosystems, Foster City, USA). The qRT-PCR conditions were as follows: 95°C for 10 min and then 40 cycles of 95°C for 30 s, 64°C for 34 s, and 72°C for 30 s. Comparisons of expression levels were determined by delta CT methods normalized to *β*-actin. The sequences for forward and reverse primers are listed in Table 1.

**Table 1:** Sequences of the primers for PCR.

Gene name	Accession number	Primer sequence (5’-3’)	Product size (bp)	
*Gclc*	XM_419910.4	GGACGCTATGGGGTTTGGAA		122
		AGGCCATCACAATGGGACAG		
*Gclm*	NM_001007953.1	CCATAGGCACCTCTGACCTTG		110
		CGGCATCACGCAACATGAAG		
*Glrx*	NM_205160.1	GAACCGTCCCTCGTGTGTTT		93
		GACGTAGCATCATGGGGAGC		
*Gpx1*	NM_001277853.1	AGTACATCATCTGGTCGCCG		137
		CTCGATGTCGTCCTGCAGTT		
*Txn*	NM_205453.1	GTGCATGCCAACATTCCAGT		118
		CTCCATGGCGGGAGATTAGAC		
*Txnrd1*	NM_001030762.2	ATGGAGCAAACAAACGTGCC		119
		CCCGCGTAAAGCCTTTGAAC		
*Nrf2*	NM_001030756.1	CTGCTAGTGGATGGCGAGAC		132
		CTCCGAGTTCTCCCCGAAAG		
*HO-1*	NM_205344.1	AGCTTCGCACAAGGAGTGTT		106
		GGAGAGGTGGTCAGCATGTC		
*β*-actin	NM_205518	ACACCCACACCCCTGTGATGAA		136
		TGCTGCTGACACCTTCACCATTC		

### Immunohistochemistry

Immunohistochemistry (IHC) was carried out following standard procedures. Briefly, antigen retrieval was performed in a 10 mM sodium citrate buffer (pH6.0) for 20 min followed by an endogenous peroxidase block using 3% hydrogen peroxide. Blocking was performed in 5% goat serum (Boster Bioengineering Co., Ltd., Wuhan, China) for 20 min at room temperature. Tissue sections were incubated overnight at 4°C with primary antibody against Nrf2 (1:200). Biotinylated secondary antibodies were used, followed by incubation with horseradish peroxidase-conjugated streptavidin. Sections were then exposed to Diaminobenzidine (DAB) to develop color. Sections were counterstained with hematoxylin for 3 min.

### Western blot

Ovarian tissue lysates were prepared using ice-cold RIPA supplemented with proteinase inhibitors and lysed for 20 min. Protein concentrations were determined using a BCA protein assay kit (Nanjing Jiancheng Bioeng Ins). 20 μg protein was loaded on SDS-PAGE gel and separated by electrophoresis and transferred to a polyvinylidene difluoride (PVDF) membrane (Millipore, Bedford, USA). After blocking, the blots were probed with corresponding primary antibodies with optimized conditions and then incubated with the secondary antibody. Immunological signals were detected by enhanced chemiluminescence (ECL) Kit (Bio-Rad, Hercules, USA) using a ChemiScope 3400 Mini machine (Clinx, Shanghai, China). The band intensities were quantified using Quantity one software and the results were normalized to β-Tubulin.

### Immunofluorescence staining

For BrdU detection, deparaffinized and rehydrated, ovarian tissue sections were first performed in a 10 mM sodium citrate buffer for 20 min at 100°C for antigen retrieval. Subsequently, the sections were denatured using 2 M HCl for 30 min at 37°C, then neutralized in 0.1 M sodium tetraborate for 10 min at room temperature. After blocking with 5% goat serum, tissue sections were incubated with mouse anti-BrdU monoclonal antibody (1:200, G3G4, DSHB, USA) overnight at 4°C, followed by incubation with goat anti-mouse secondary antibody (1:500) conjugated to TRITC (Invitrogen, Carlsbad, USA) for 1 h at 37°C. The sections were subsequently stained with 4’,6-Diamisino-2-phenylindole (DAPI, Sigma-Aldrich) for cell nuclei and imaged on a fluorescence microscope. The number of BrdU positive cells (red) was counted and expressed as a percentage of the BrdU labeling cells over the total number of ovarian cells within the same fields (BrdU index).

### TUNEL analysis

The apoptosis of cells was detected using a TUNEL Brightgreen Apoptosis Detection Kit (Vazyme, Nanjing, China) according to the manufacture’s instruction. For the calculation of TUNEL index, five fields of each section were randomly selected for counting the number of the TUNEL positive cells (green), and the apoptosis index was calculated as the percentage of the green labeling cells over the total number of ovarian cells (TUNEL index).

### Statistical analysis

All experiments were repeated at least three times. Data were analyzed by one way ANOVA with post hoc Dunnett’s test and independent samples *t*-test using the SPSS 20.0 software (SPSS Inc., Chicago, USA) and presented as mean±s.e. Results were considered statistically significant at *P* < 0.05.

## Supplementary Material

Supplementary Figures
