# Supplementary material for: Lycopene ameliorates oxidative stress in the aging chicken ovary via activation of Nrf2/HO-1 pathway
Source: Aging (Albany NY). 2018 Aug 16;10(8):2016–36. doi: 10.18632/aging.101526 (PMC6128425; doi:10.18632/aging.101526)
Supplement: Supplementary Figures [file aging-10-101526-s001.pdf]

SUPPLEMENTARY MATERIAL

The D-gal induced damage of ovarian tissue morphology, the inhibition of cell proliferation, and the promotion of cell apoptosis, all exhibited a dose-dependent increase. The results of Masson staining showed that after treatment with 2.5 mg/mL D-gal for 72 h, the degree of ovarian tissue fibrosis was similar to that in D580 ovarian tissues. In addition, the antioxidant capacity of ovarian tissues treated with 2.5 mg/mL D-gal decreased to the level of the D580 ovaries. Based on the evaluation of tissue morphology, cell proliferation and apoptosis rates [17], tissue fibrosis and antioxidant capacity, the dose of 2.5 mg/mL D-gal was chosen as the optimal concentration in the subsequent experiments (Supplementary Figure 1). Likewise, four gradient concentrations of lycopene (from 1 ng/mL to 1000 ng/mL) were screened for the optimal concentrations under D-gal-induced damage. Lycopene, at the two concentrations (100 and 1000 ng/mL), significantly reduced the ovarian tissue damage induced by D-gal. Based on the tissue morphology, cell proliferation and apoptosis rates, 100 ng/mL of lycopene was selected as the optimal concentration in the following experiments (Supplementary Figures 2-4).

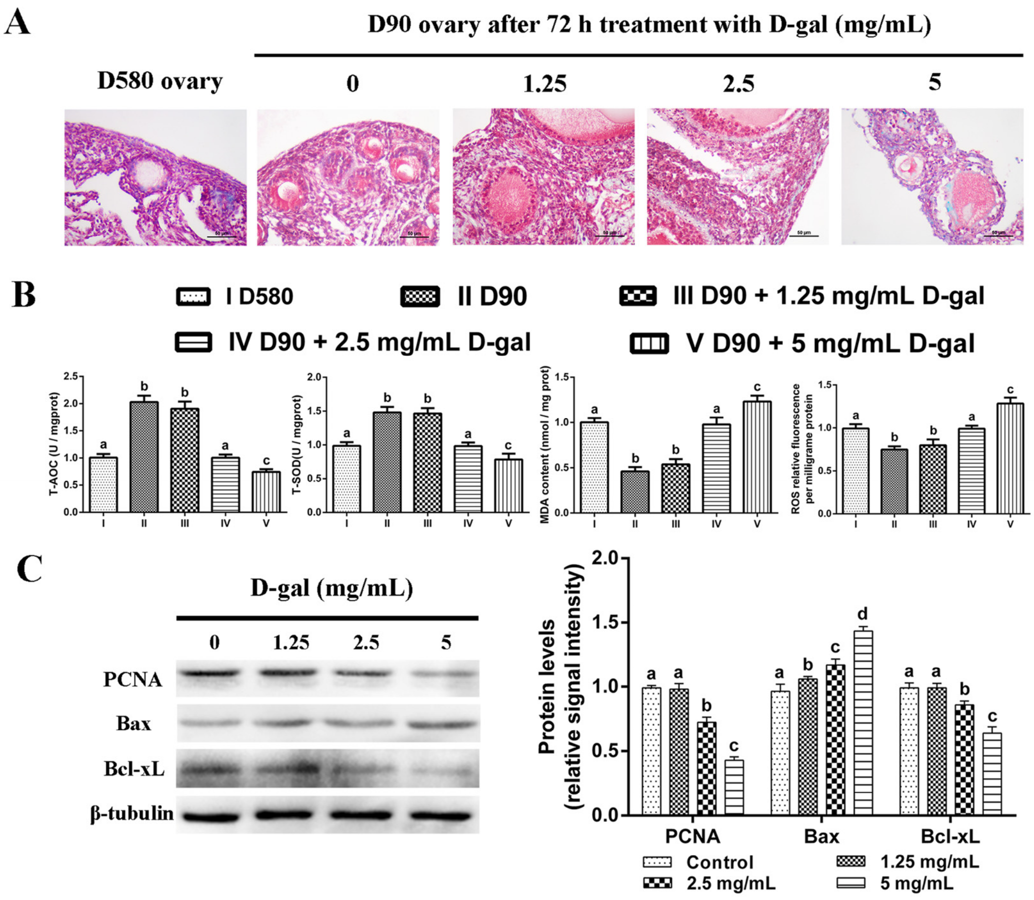

Supplementary Figure 1. Effects of D-gal on ovarian tissue fibrosis, antioxidant capacity, cell proliferation and apoptosis. (**A**) Representative morphology of D580 ovarian tissues and ovarian tissues after 72 h of culture in control and the 1.25-5 mg/mL D-gal group, performed by Masson staining, scale bar: 50 μm. (**B**) Levels of T-AOC, T-SOD, MDA and ROS in D580 ovarian tissues and ovarian tissues after 72 h of culture in control, 1.25-5 mg/mL D-gal group. (**C**) Expression levels of PCNA, Bax and Bcl-xL in ovarian tissues after 72 h of culture in control and 1.25-5 mg/mL D-gal groups.

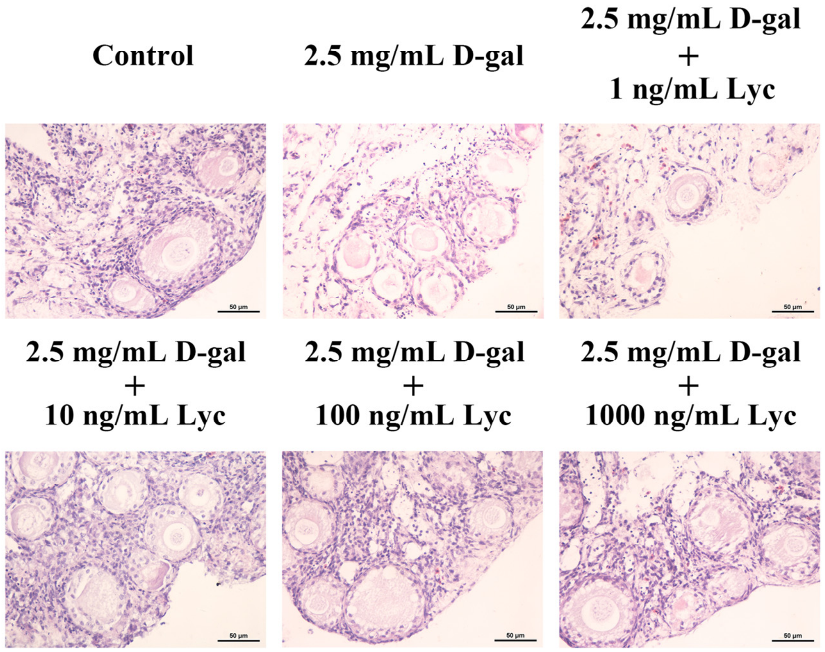

Supplementary Figure 2. Protective effect of lycopene on D-gal-induced ovarian morphological change by HE staining. Scale bar: 50 µm.

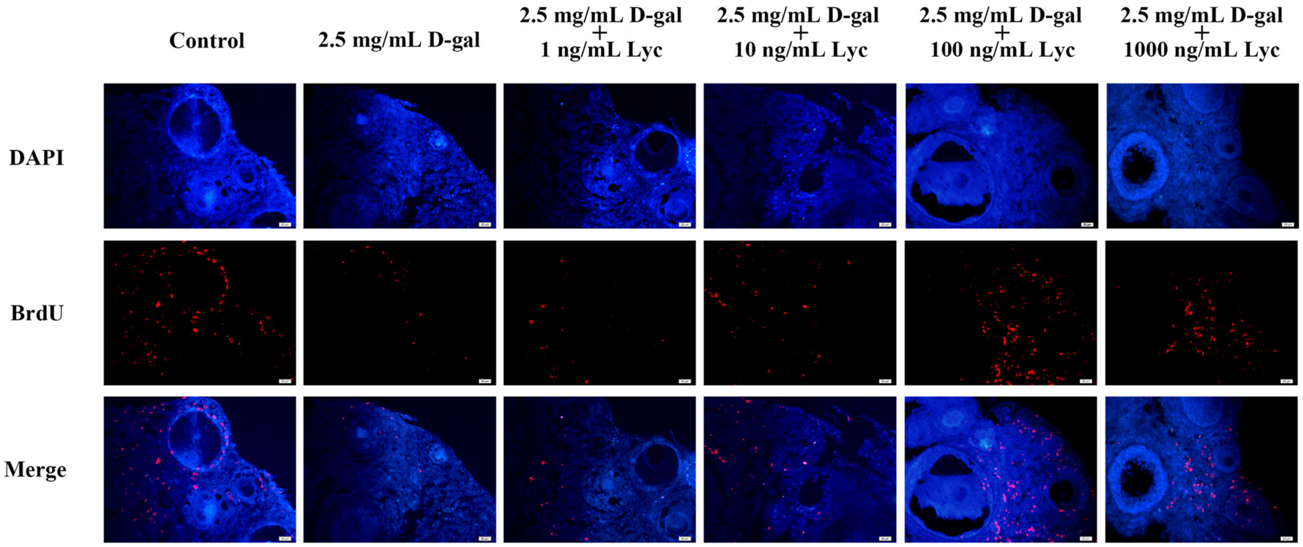

Supplementary Figure 3. Protective effect of lycopene on D-gal-induced ovarian cell proliferation decline as revealed by BrdU staining. DAPI staining was performed to stain the nucleus. Scale bar: 20 µm.

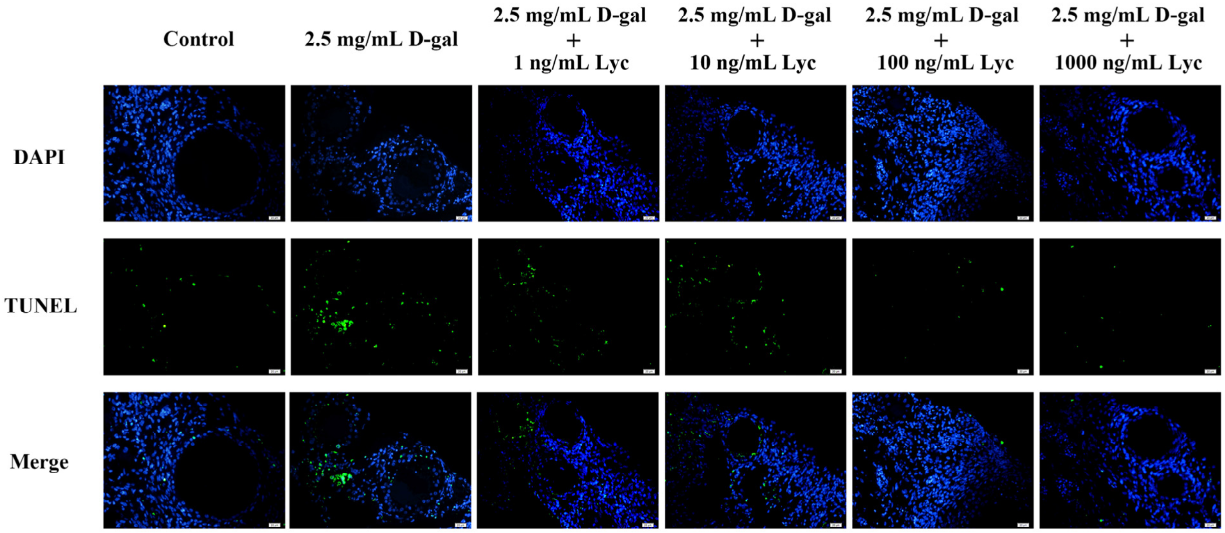

Supplementary Figure 4. Protective effect of lycopene on D-gal-induced ovarian cell apoptosis by TUNEL assay. DAPI staining was performed to stain the nucleus. Scale bar: 20 μm.
